# Tracking oocyte development and the timing of skipped spawning for north‐east Arctic haddock ( *Melanogrammus aeglefinus* )

**DOI:** 10.1111/jfb.15057

**Published:** 2022-04-20

**Authors:** Frida Tronbøl, Edda Johannesen, Maud Alix, Thassya C. dos Santos Schmidt, Katerina Charitonidou, Arild Folkvord, Olav Sigurd Kjesbu

**Affiliations:** ^1^ Department of Biological Sciences University of Bergen Bergen Norway; ^2^ Institute of Marine Research Bergen Norway; ^3^ Marine and Freshwater Research Institute Neskaupstaður Iceland; ^4^ Department of Biology Aristotle University of Thessaloniki Thessaloniki Greece

**Keywords:** atresia, Barents Sea, haddock, histology, oocyte development, skipping

## Abstract

The present study tracked oocyte development over 9 months and noted incidences of ‘skipping’, *i.e.*, adults terminating their upcoming reproductive cycle, in field‐caught north‐east Arctic (NEA) haddock (*Melanogrammus aeglefinus*), currently the largest stock of this species. Applications of advanced image and histological techniques revealed the presence of cortical alveoli oocytes (CAO), which prevailed as the most advanced oocyte phase for 4–5 months. This new finding of an extended and early appearance of CAOs in this gadoid was supported by that vitellogenesis first started to appear 3 months later. The subsequent oocyte growth trajectories indicated that larger individuals [total length (TL) = 70 cm] typically spawn in the order of 3 weeks earlier than the smaller ones (TL = 40 cm). The spawning season appeared stretched over about 3 months. The majority of skipping females arrested oocyte growth at the CAO phase followed by atretic reabsorption. Compared to those individuals maturing for the spawning season, ‘skippers’ generally exhibited lower body condition, characterized also by relatively lower liver sizes at the time of the main spawning season. This study demonstrated well‐developed skipping dynamics, but also that the CAO period, *i.e.*, when skipping takes place, may be exceedingly long in this commercially valuable gadoid and that its reproductive cycle in many ways deviates from that of the data‐rich, sympatric NEA cod (*Gadus morhua*).

## 1 INTRODUCTION

**FIGURE 1:**
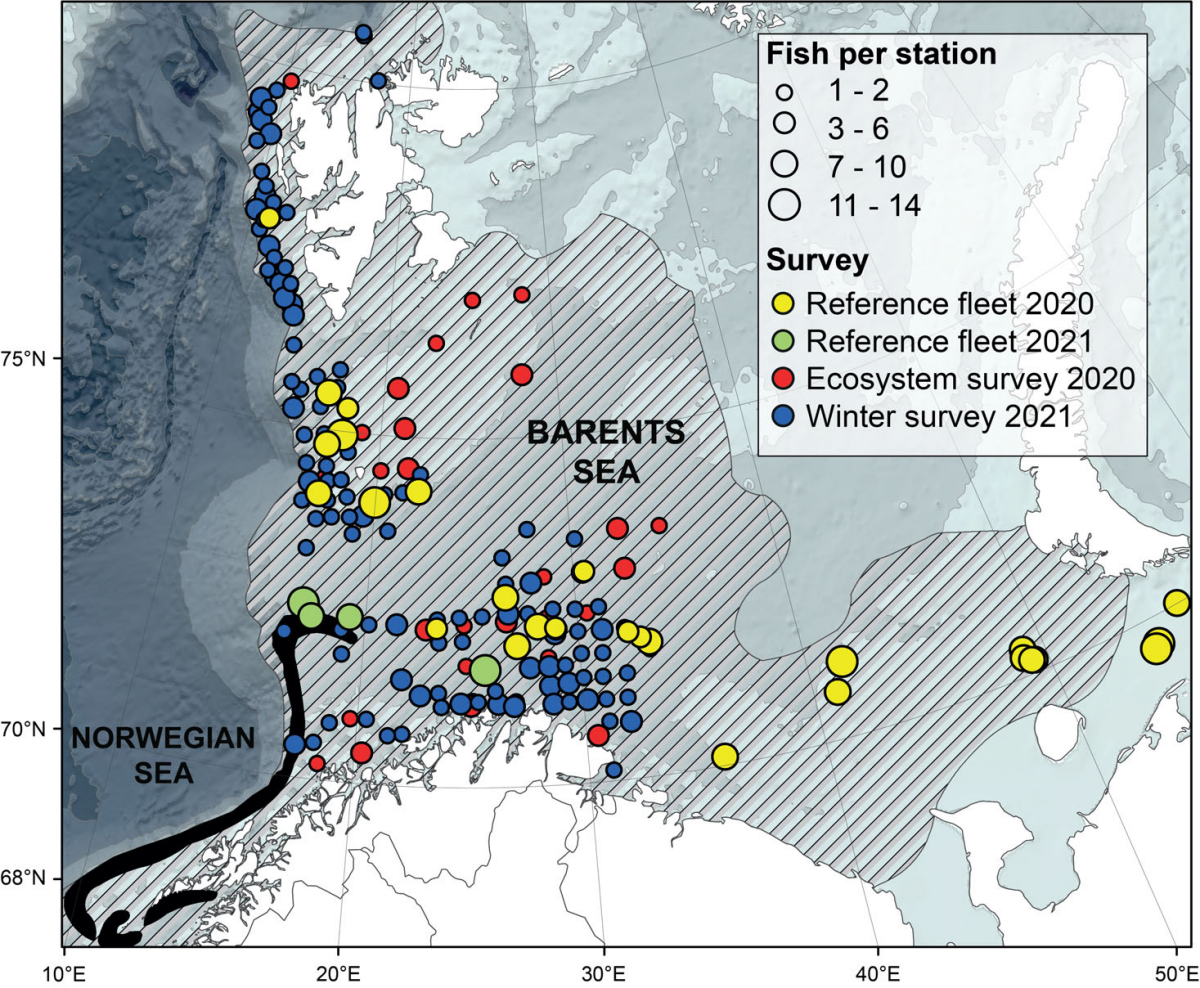
Map of sampling stations in the Barents Sea. The size of the bullets is proportional to the number of individuals sampled at each location. The colours of the bullets correspond to the surveys involved in the sampling collection. *Melanogrammus aeglefinus* (NEA stock) distribution (hatched area) follows the shelf break and the extent of Atlantic water flowing into the Barents Sea from the south‐west. The main spawning grounds are represented in black, modified from the official *M. aeglefinus* (NEA stock) map from the Institute of Marine Research, Norway (Sivle & Johnsen, 2016)

The reproductive success of individuals largely defines the degree to which a population can replenish and persist over time. Thus, both the total egg production and the processes affecting the survival of early life stages are vital factors (Lowerre‐Barbieri *et al*., 2011; Trippel, 1999). Haddock (*Melanogrammus aeglefinus*; L. 1758) is a commercially important demersal gadoid distributed on both sides of the north Atlantic. The largest stock of this species—north‐east Arctic (NEA) hereafter referred to as *M. aeglefinus* (NEA stock)—resides in the Barents Sea (Johannesen *et al*., 2019) whereas its management unit area extends south to 62°N, although a recent genetic study suggests a genetic break at 68°N (Berg *et al*., 2021) (Figure 1). As for *M. aeglefinus* in general, the oocyte development and maturity dynamics of this stock have so far been addressed within restricted time windows, *i.e*., at or near the spawning season (Skjæraasen *et al*., 2013, 2015). Hence, central aspects of its reproductive cycle still require closer attention, as addressed below.

As an iteroparous species, *M. aeglefinus* is assumed to follow an annual reproductive cycle. A growing body of evidences, however, suggests that in some years more than half of the sexually mature portion of *M. aeglefinus* (NEA stock) does not follow this pattern (Skjæraasen *et al*., 2015). This phenomenon—known as skipped spawning, *i.e*., adults terminating their upcoming reproductive cycle—has the potential to bias the spawning stock biomass (SSB) if not accounted for in assessment models (Rideout & Tomkiewicz, 2011). Although skipping individuals of *M. aeglefinus* (NEA stock) are excluded from annual SSB estimates, uncovering under what conditions insufficient energy reserves or associated trade‐offs (Skjæraasen *et al*., 2020) are controlling interruption of the reproductive cycle would help to improve the prediction of the stock's response to environmental stressors.

**FIGURE 2:**
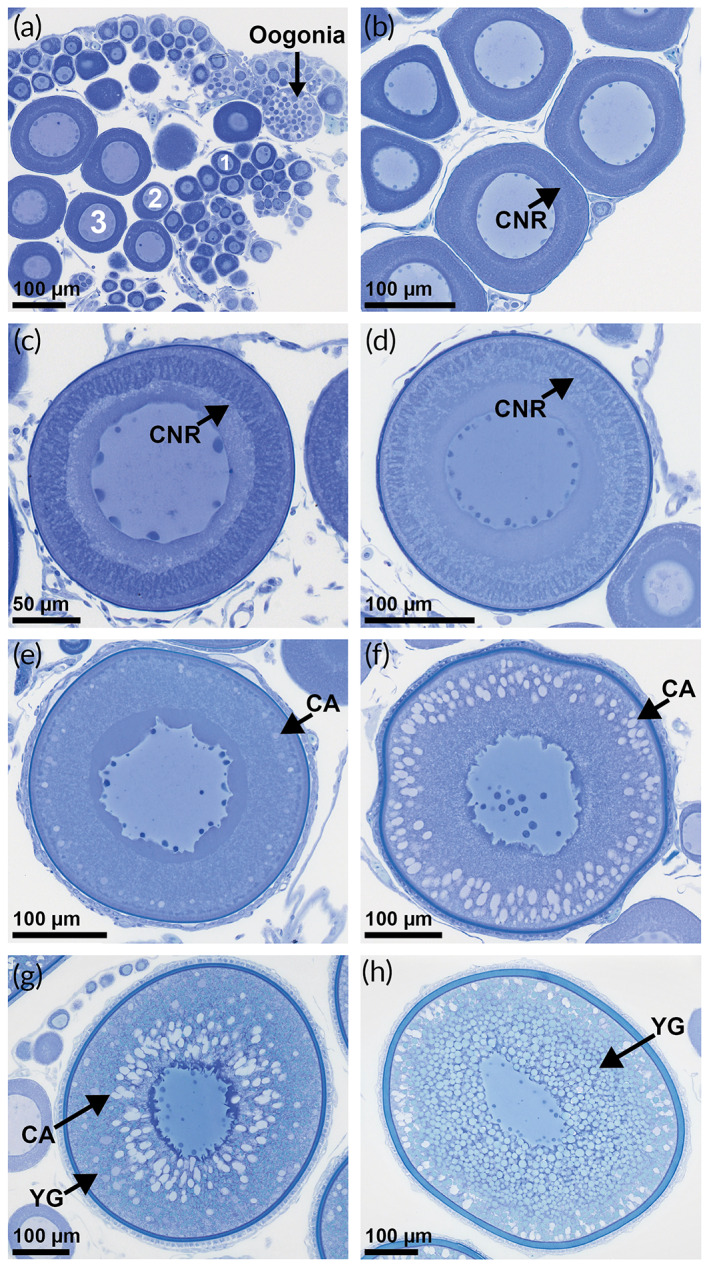
Histological sections of the studied oocyte development phases of *Melanogrammus aeglefinus* (NEA stock) stained with toluidine blue. (a) First developmental phases, oogonia and PVO phases 1, 2 and 3, cytoplasm is uniform and homogenous. (b) PVO phase 4A, a circumnuclear ring (CNR; arrow) appears in the cytoplasm as an indistinct feature located centrally. (c) PVO phase 4B, the CNR has become distinct and has migrated towards the periphery of the cytoplasm. (d) PVO phase 4C, the CNR is gradually disappearing. (e) ECAO phase, commences when cortical alveoli (CA) appear at the periphery of the cytoplasm. (f) LCAO phase, CA increases in size and quantity, and the chorion becomes more pronounced. (g) EVO phase, small yolk granules (YG) appear on the periphery of the cytoplasm. (h) LVO phase, an increase in number, size and distribution of the yolk granules, which occupy virtually all the cytoplasm

**FIGURE 3:**
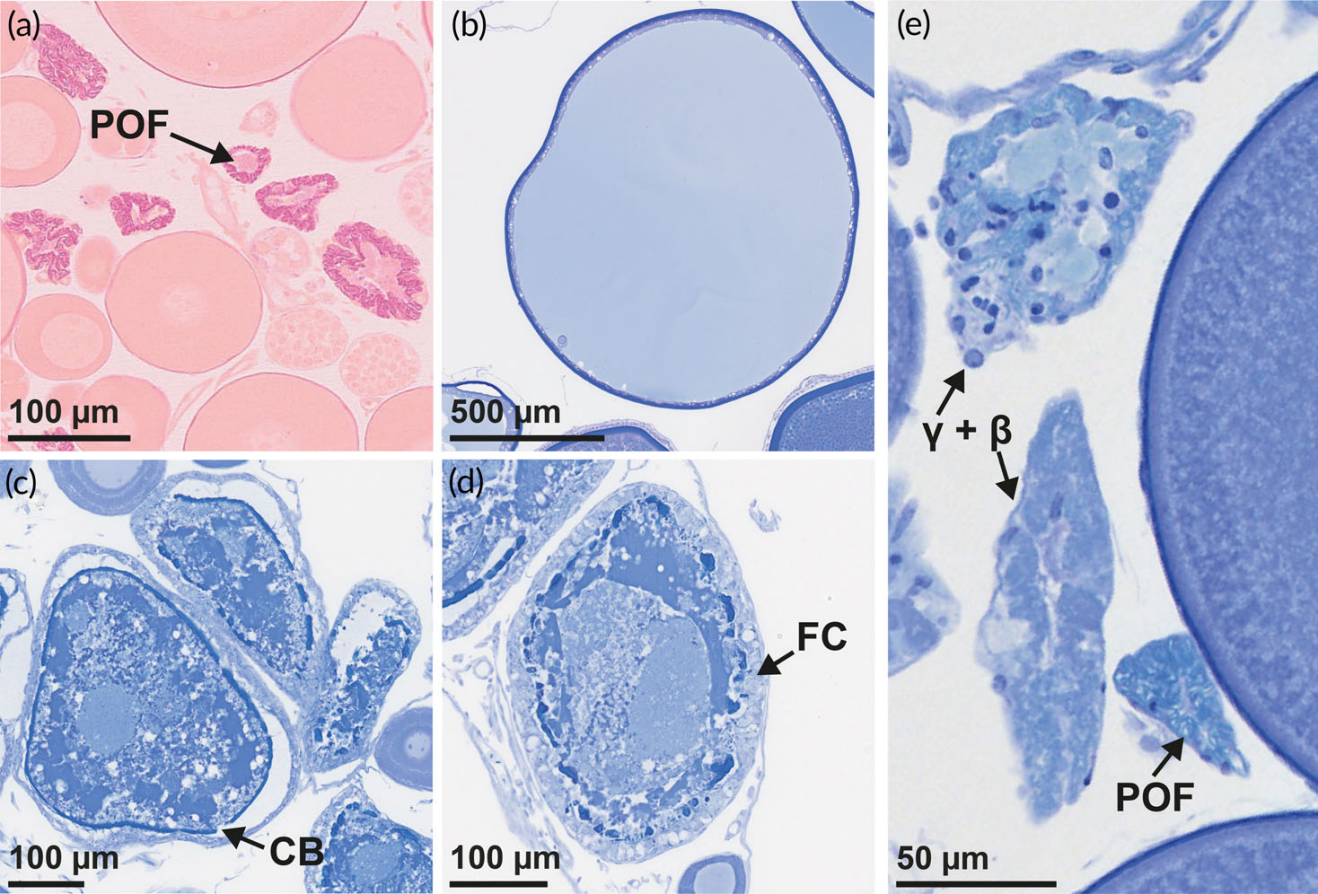
Histological sections of hydrated oocytes, postovulatory follicles and degenerating oocytes in *Melanogrammus aeglefinus* (NEA stock). (a) Post‐ovulatory follicles (POF) stained with periodic acid‐Schiff (PAS) and Mallory trichrome. (b) Hydrated oocyte with cytoplasm fully displaced towards the periphery. (c) Early alpha atresia, breaks are visible in the chorion (CB). (d) Late alpha atresia, chorion appears fragmented whereas follicle cells (FC) have multiplied in numbers. (e) Combined beta and gamma atresia (β + γ), chorion has disintegrated and yolk granules are absent. Slides in (b), (c), (d) and (e) were stained with toluidine blue

It is generally accepted that the presence of cortical alveoli oocytes (CAO), or advanced previtellogenic oocytes (PVO) (Kjesbu *et al*., 2011) (Figure 2), is the first marker that the female under scrutiny is going to spawn in the forthcoming spawning season (Skjæraasen *et al*., 2010). The reproductive cycle of some females may, however, get arrested at this advanced PVO phase (resting skipper) (Rideout *et al*., 2005). Alternatively, oocytes might be reabsorbed at the early CAO phase, a process which has been associated with insufficient energy reserves (reabsorbing‐CAO skipper) (Rideout & Tomkiewicz, 2011; Skjæraasen *et al*., 2009, 2015). Conversely, maturing *M. aeglefinus* (NEA stock) which continue oocyte development will migrate southwards to spawning grounds (~68 to 72°N) along the shelf break from the Lofoten archipelago and northwards (Sivle & Johnsen, 2016) in January ‐ February (Bergstad *et al*., 1987; Sætersdal, 1952). However, some of these individuals may still interrupt their reproductive cycle by reabsorbing all vitellogenic oocytes (VO) (Figure 2) through follicular atresia (reabsorbing‐VO skippers) (Figure 3) or even retain their fully ripened eggs (retaining skippers) (Rideout & Tomkiewicz, 2011).

Although various aspects of the reproductive dynamics of *M. aeglefinus* do exist in the literature, no studies have dedicatedly tracked oocyte development following photoperiod cues in summer (Davie *et al*., 2007) and autumn (Kjesbu *et al*., 2010), *i.e*., when processes associated with the fish's energetic state may be important for the reproductive decision (see above). We took advantage of modern image analysis techniques in combination with histology to pin‐point the time course of reproductive events in field‐caught *M. aeglefinus* (NEA stock) through the initiation of the reproductive cycle until the onset of spawning to (i) examine oocyte development and timing of reproductive commitment, (ii) investigate the dominant mode and fundamental mechanisms determining skipping, and, finally, (iii) discuss and compare our findings to related studies on the sympatric *Gadus morhua* (L.) (NEA stock), a group‐synchronous and determinate batch spawner like *M. aeglefinus* (Murua & Saborido‐Rey, 2003).

## 2 MATERIALS AND METHODS

### 2.1 Biological sampling and biometric indices

*M. aeglefinus* (NEA stock) were collected in the Barents Sea at 181 sampling locations during autumn, winter and spring (Figure 1). Among these sampling locations, 93 stations were represented by only one (72) or two fish (21) (Figure 1). The samples were either collected during statutory research surveys (demersal trawl) or by the Norwegian Reference Fleet (long line, Danish seine or demersal trawl) (Table 1). Any gear selectivity was left unconsidered in this individual‐based study. In accordance with standard fishing practices, all animals were deceased at landing. As such, animal ethics approval for this project was not required.

**TABLE 1:** Summary of biological sampling of NEA haddock (*Melanogrammus aeglefinus*) in the Barents Sea

Time period	Survey type	Survey	Stations	*n*(H)	*n*(IA)
Aug to Sep (2020)	Fisheries independent	Ecosystem Survey, IMR	31	35	70
Aug (2020) to Apr (2021)	Fisheries dependent	The Norwegian Reference Fleet	32	172	280
Jan to Mar (2021)	Fisheries independent	Winter Survey, IMR	118	91	240

*Note*: Ovary tissue and biometric information were collected monthly by commercial vessels from the Norwegian Reference Fleet (Moan *et al*., 2020) and additionally, from two annual surveys run jointly by the Norwegian Institute of Marine Research (IMR) and the Polar Branch of the Russian Federal Research Institute of Fisheries and Aquaculture (PINRO): the Joint Norwegian‐Russian Ecosystem Survey in the Barents Sea (Ecosystem Survey; van der Meeren & Prozorkevich, 2020) and the Joint Norwegian‐Russian Trawl‐Acoustics Survey for demersal fish (Winter Survey; Fall *et al*., 2020). *n*: number of fish samples processed with histology (H) and whole mount [image analysis (IA)].

Data collected for all fish included total length (TL, 1 cm), whole weight (*W*, 1 g), ovary weight (OW, 0.01 g) and liver weight (LW, 0.01 g). The currently applied equations for Fulton's condition factor (*K*) and somatic hepatosomatic index (HSI_S_) were given as *K* = 100 × *W*/TL^3^ and HSI_S_ = 100 × LW/(*W* − OW) (Skjæraasen *et al*., 2009). The *W vs*. TL power function relationship was based on isometry (one‐sample *t*‐test, d.f. = 588, *P* = 0.25); the 95% confidence interval of the exponent parameter ranged between 2.98 and 3.05. Ages were obtained from otoliths (Mjanger *et al*., 2019).

Just after completion of fish handling, an ovarian subsample (~2 g) was excised from the mid‐section of the right lobe (assuming homogeneity; Kjesbu & Holm, 1994) and placed in a BiopSafe container (Axe‐lab, Denmark, https://www.axlab.dk/) with 20 ml of 4% formaldehyde.

### 2.2 Laboratory analysis

#### 2.2.1 Image analysis (whole mount)

All ovarian samples presently considered (*n* = 590) were subjected to digital image analysis according to the auto‐diametric method (Thorsen & Kjesbu, 2001). The initial step in this protocol included the use of an ultrasonic pen on a pipetted subsample to separate oocytes, which were stained with 2% toluidine blue and 1% sodium tetraborate, and, finally, photographed under a dissecting microscope, *i.e*., using similar laboratory equipment and protocols as detailed earlier (Anderson *et al*., 2020). The diameter of at least 200 oocytes (to the nearest μm) were measured using open‐source ImageJ software (v. 1.52, https://imagej.nih.gov/ij/) with the plugin ObjectJ (https://sils.fnwi.uva.nl/bcb/objectj/). The mean size of the 20 largest oocytes, hereafter labelled as oocyte leading cohort diameter (LC, in μm), reflected the female maturity stage (Thorsen & Kjesbu, 2001).

#### 2.2.2 Histology

Ovarian subsamples (*n* = 298) were analysed histologically to detail microscopic structures—oocyte development phases as well as post‐ovulatory and atretic follicles (Figures 2 and 3)—to aid highly precise maturity staging (see below). These pieces of ovarian tissue were processed using standard protocols for resin embedding (Kulzer, Technovit 7100, Wehrheim, Germany), producing two sets of 4 μm sections stained with 2% toluidine blue and 1% sodium tetraborate or, alternatively, periodic acid‐Schiff (PAS) and Mallory trichrome stain to further document any presence of post‐ovulatory follicles (POF, Figure 3a) (Witthames *et al*., 2010). During the subsequent screening of sections, PVO (late primary growth oocytes) were divided according to Shirokova (1977) into four phases (PVO1, PVO2, PVO3 and PVO4) (Figure 2), and the latter phase was additionally split into three subphases (4A, 4B and 4C) contingent on the shape and location of the circumnuclear ring (CNR) (Figure 2b–d). Secondary oocyte growth included early (ECAO) and late cortical alveoli (LCAO), early (EVO) and late vitellogenic (LVO) phases (Figure 2), examining oocyte size, zona radiata (chorion) appearance and the extent of cortical alveoli and yolk granules (Serrat *et al*., 2019; Skjæraasen *et al*., 2009). Final oocyte maturation was represented by hydrating‐ovulated oocytes (HO) (Figure 3b). The intensity of atresia (as a percentage) was given as the number of atretic oocytes divided by the sum of atretic and normal oocytes in the most advanced phase. The type of atresia was classified by the degree of breakdown of the chorion and, if necessary, along with any presence or absence of yolk: early alpha (Eα), indicated by small breaks in the chorion (Figure 3c); late alpha (Lα), seeing fragmentation of the chorion (Figure 3d); a combined beta and gamma (β + γ) type, characterized by disintegration of the chorion, no visible yolk content and significantly reduced follicle size (Figure 3e) (Witthames *et al*., 2010). For the present study, atresia values were thereafter grouped as very low (<5%), low (5%–25%), moderate (25%–50%) and massive (>50%).

Based on detailed examinations of histological slides, immature, skipping, maturing or developing (Brown‐Peterson *et al*., 2011), spawning and spent maturity stages (categories) were derived from the most advanced oocyte phase, any presence of POF and the intensity of atretic oocytes. An obligatory feature of ‘skippers’ was either (i) the existence of POF along with PVO (resting skippers) or (ii) the existence of POF along with CAO or VO undergoing atresia, *i.e*., reabsorbing‐CAO and reabsorbing‐VO skippers, respectively (see Introduction).

### 2.3 Statistical analysis

Prior to performing any test on changes in LC over time, the date variable was converted into the number of days, where the earliest sampling day, August 14, 2020, was coded as day 1, and subsequent sampling days were given a number past this day, *i.e*., the final sampling day, April 30, 2021, was coded day 260. To provide the estimated time of spawning, a multiple linear regression was run, where LC was treated as a continuous response variable, and day and TL were treated as continuous predictors, initially considering also the contribution of *K* and HSI_S_ (see below) as well as measuring the extent of multicollinearity (variance inflation factors, VIF). In this exploratory analysis, the minimum value of LC was set at 450 μm to ensure that all females included were *de facto* maturing (see below), whereas the maximum LC value was set at 800 μm assuming imminent spawning (Alekseyeva & Tormosova, 1979). A two‐way analysis of variance (ANOVA) with Tukey's HSD (honestly significant difference) *post hoc* test addressed any significant difference in TL and age between microscopic (histological) maturity categories. Finally, a two‐way ANOVA was used to examine if the continuous response variables *K* and HSI_S_ varied between maturity categories at different sampling months, including an interaction term (*maturity category × month*).

Model assumptions were assessed by residual‐fit plots and tested for normality and homogeneity of variance using Shapiro–Wilk and studentized Breusch–Pagan tests. When parametric violations were detected, data were arcsine transformed in the case of ratios represented by *K* and HSI_S_. Finally, Tukey's HSD *post hoc* test was used to examine differences detected by ANOVA. All analyses were performed in R (R Core Team, 2020) (version 4.0.2). For data tidying, plotting and statistics the following R packages were used: tidyverse (Wickham, 2017), ggpubr (Kassambara, 2018), viridis (Garnier, 2018), multcomp (Hothorn *et al*., 2008) and emmeans (Lenth, 2021). Values are presented as mean ± standard deviation (s.d.) and rejection of the null hypothesis was always set at *P* < 0.05.

## 3 RESULTS

### 3.1 Growth in leading cohort size (whole mount)

**FIGURE 4:**
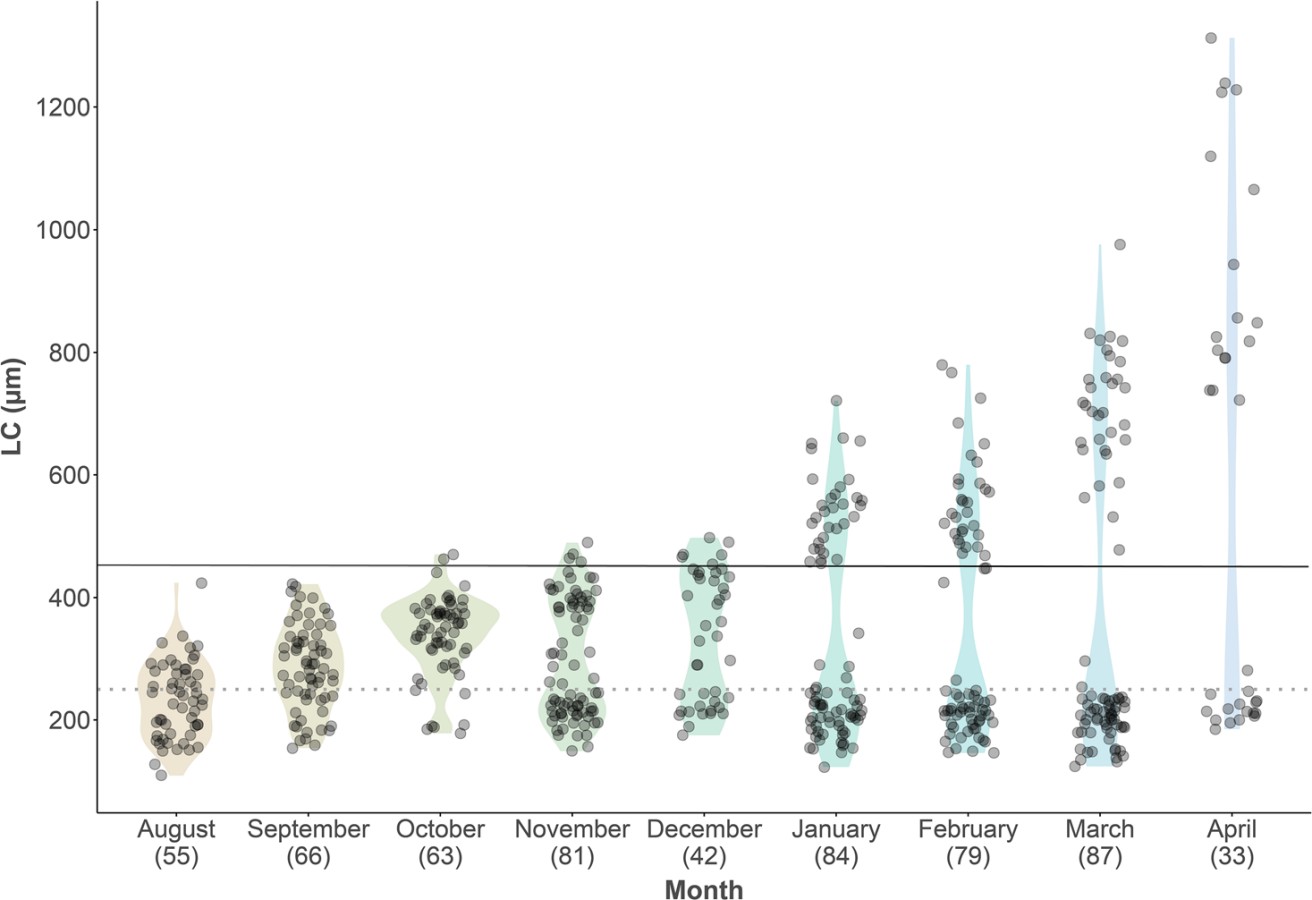
Monthly development of the oocyte leading cohort (LC) prior to the onset of spawning in *Melanogrammus aeglefinus* (NEA stock) (*n* = 590). Transparent circles and the dotted line at 250 μm represent the individual sample points and the threshold between previtellogenic and cortical alveoli oocytes, respectively. Individuals located above the solid line at 450 μm (vitellogenic oocytes) were used to estimate spawning time. The numbers in brackets denote the number of observations per month

LC of maturing individuals continued to increase over the entire study period (Figure 4). This oocyte growth resulted in the formation of a gap between PVO (set at 250 μm) and developing oocytes in October, becoming increasingly conspicuous by November, clearly separating this trajectory from the one belonging to nondeveloping females (Figure 4). The LC of maturing females increased from 292 ± 37 μm in August to 1123 ± 159 μm in April at spawning.

### 3.2 Estimated start of spawning (whole mount)

**FIGURE 5:**
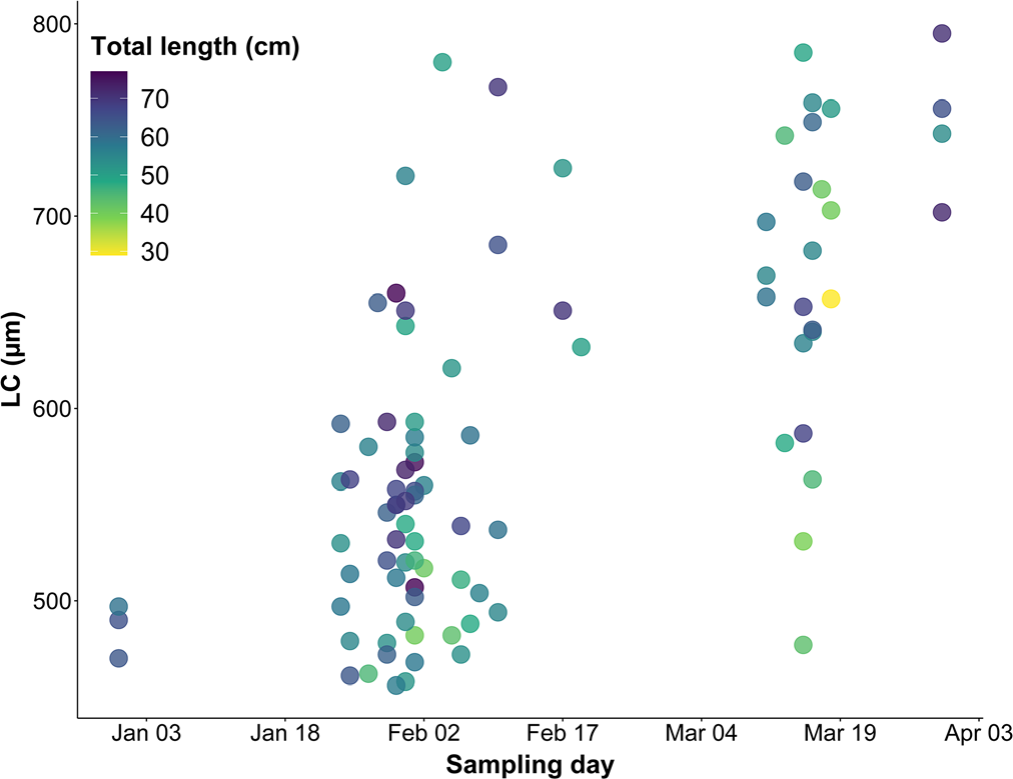
Oocyte leading cohort (LC) over time in relation to female total length in *Melanogrammus aeglefinus* (NEA stock). Only females collected from December 31, 2020 to March 30, 2021 were included in the model. Minimum LC size was set to 450 μm and maximum at 800 μm, the size at which the fish is assumed start to spawning

Substantial individual variability in LC existed at a given sampling date for fish with LC ≥450 μm, reflecting an extensive spawning season (multiple regression, *F*_4,88_ = 25.4, *r* ^2^ = 0.515, *P* < 0.001) (Figures 4 and 5). Both *K* and HSI_S_ showed an insignificant contribution (*P >* 0.05) when combined with day (*P* < 0.001) and TL (*P* = 0.04). Hence, the resulting equation with LC as response variable became:

(1) $$\mathrm{LC}=-95.8+3.07\times \mathrm{day}+2.08\times \mathrm{TL}\;\left(r^{2}=0.508,\;P<0.001\right)$$

with the separate *P* values of day and TL being <0.001 and 0.011, respectively. The mean estimated LC growth rate of 3.07 μm/day (*F*_2,90_ = 48.52, *P* < 0.001) indicated individual variations in the start of spawning of at least 3 months: on March 15 the LC in one female was 477 μm, while it was 785 μm in another, *i.e*. a difference of 308 μm, corresponding to 100 days if the LC growth rate is 3.07 μm/day.

### 3.3 Oocyte development (histology)

**FIGURE 6:**
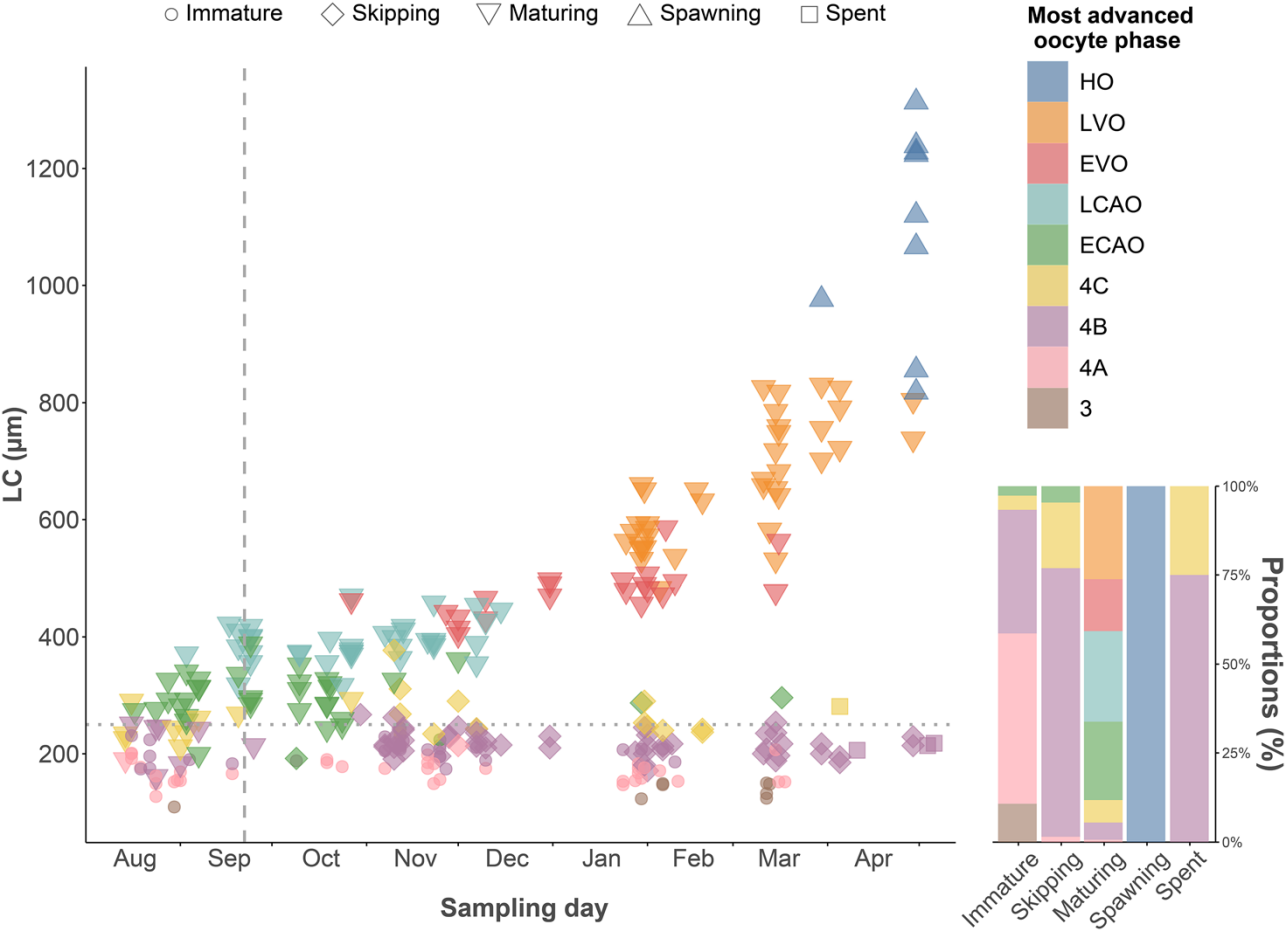
Development of the oocyte leading cohort (LC) during the reproductive cycle (August 14, 2020 to April 30, 2021) in relation to the most advanced oocyte phase in *Melanogrammus aeglefinus* (NEA stock) (*n* = 298). Colours and shapes represent the phases of oocyte development and histological maturity categories. The horizontal dotted line and the vertical dashed line denote the threshold between previtellogenic and cortical alveoli oocytes (250 μm), and the time of autumn equinox (set at September 22), respectively

Most (67 out of 75) of the studied immature females did not show any sign of oocyte development, remaining in PVO 4A (Figure 2b), with LC <250 μm (Figure 6), whereas the subsequent oocyte growth of maturing females ended with large LCs, represented by HO (Figure 3b) in April (Figure 6). The earliest signs of commitment to maturation, *i.e*., presence of CAO, were observed in August: four out of 13 females had at that time reached ECAO (Figures 2e and 6). By September, 24 out of 31 females had progressed to ECAO and LCAO (Figures 2f and 6). Approximately 5 weeks following the autumn equinox (September 22), yolk granules appeared in the cytoplasm of a 62 cm female (age 6 years), entering EVO (Figure 2g) at LC 462 μm, while most females in October (22 of 24) possessed either ECAO or LCAO (Figure 6). In November, LCAO dominated (11 of 13), the two other studied females being in either ECAO or EVO. By December, the EVO phase was dominant (eight out of 14), with the remaining six females still possessing ECAO and LCAO (Figure 6). The cortical alveoli phases were no longer present in January and February as all maturing females had transitioned into vitellogenesis, either EVO or LVO (Figure 2h) (January 10 of 14, February seven of 12) (Figure 6). By March, LVO was even more dominant (16 out of 19) (Figure 6) and LC (710 ± 86 μm) showed an abrupt increase compared to the previous month. The first spawning female was observed at the end of the month, possessing HO (Figures 3b and 6). By April, eight females (out of 13) showed HO, varying from 818 to 1313 μm (Figure 6). The remaining females possessed LVO at LC = 776 ± 44 μm. Five of those females in LVO in March and April had LC >800 μm (821 ± 10 μm) (Figure 6). All four females identified as spent were collected in April (Figure 6), characterized by abundant POF (Figure 3a) and atretic follicles (Figure 3c,d).

### 3.4 Skipping modes (histology)

**FIGURE 7:**
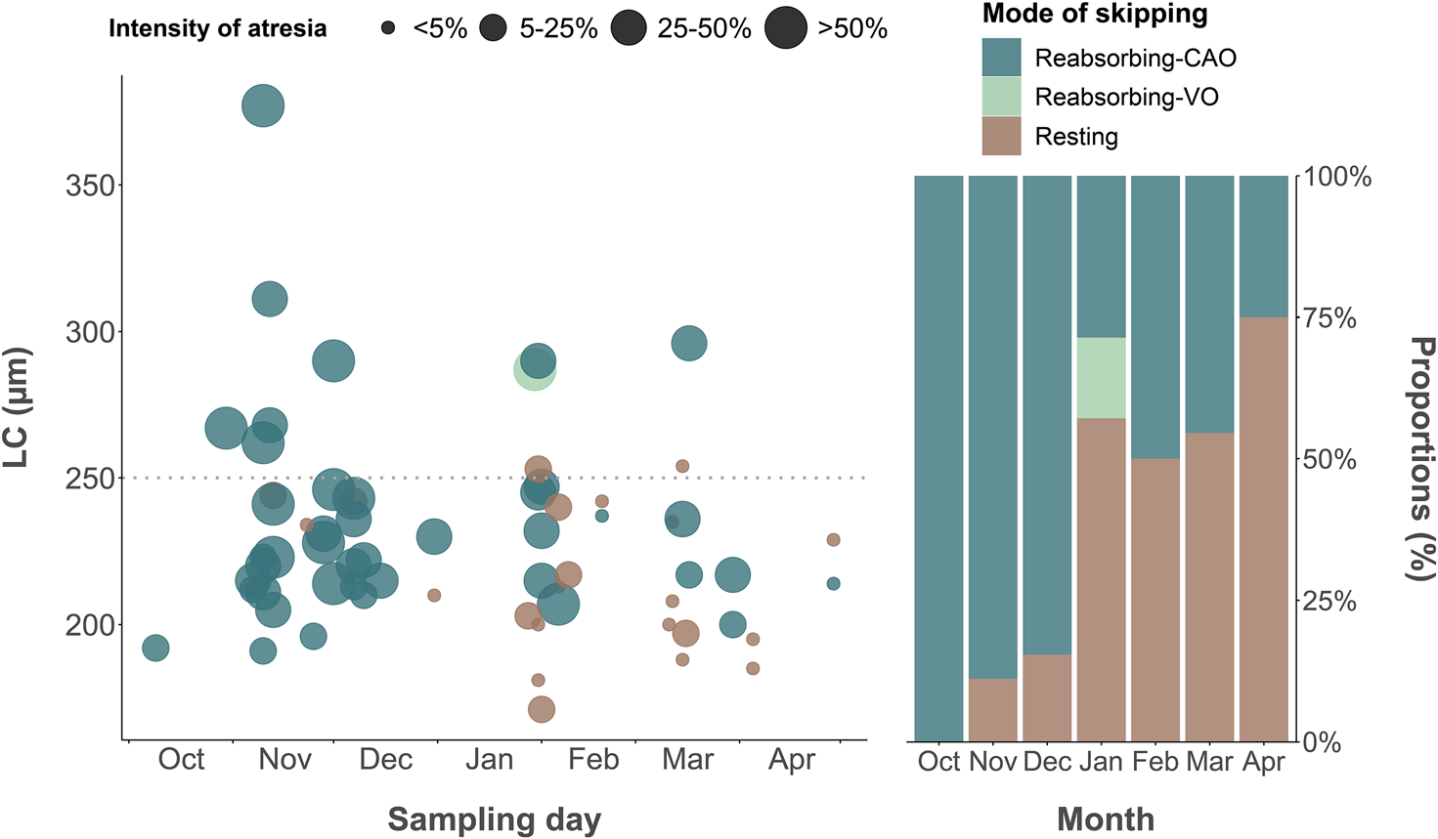
Oocyte leading cohort (LC) size over time in relation to the intensity of atresia observed in reabsorbing cortical alveoli (reabsorbing‐CAO), reabsorbing vitellogenic oocyte (reabsorbing‐VO) and resting skippers (*n* = 65) in *Melanogrammus aeglefinus* (NEA stock). The size of the bullets is proportional to the group‐classified intensity of atresia while the colours represent the mode of skipping

The first skipping female was identified (Figure 3a) at the end of October by the presence of massive atresia (>50%) of ECAO (Figure 7). From November onwards, the occurrence of skippers became much more frequent and was observed in all subsequent months. In total, 65 skipping females were identified during the present sampling period. Of these, 42 were identified as reabsorbing‐CAO skippers, exhibiting reabsorption of nearly all CAO, and 22 were classified as resting skippers by the presence of advanced PVO, with very low atretic intensity (<5%) (Figure 7). Only one female was identified as a reabsorbing‐VO skipper, possessing massive atresia of all VOs (Figure 7). The vast majority of skipping females possessed both Lα (Figure 3d) and β + γ atresia (Figure 3e).

### 3.5 Biometric and age differences by maturity category (histology)

**FIGURE 8:**
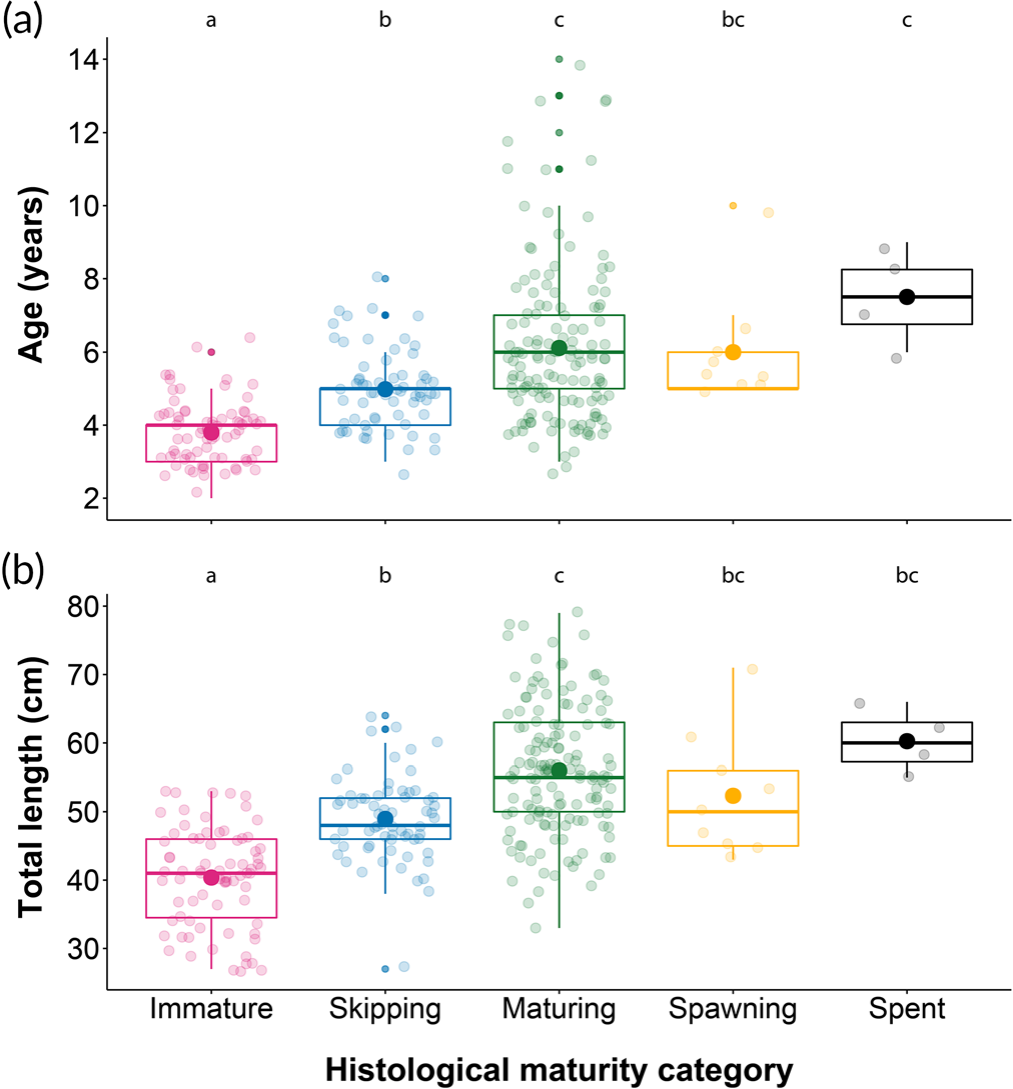
The proportion of immature, skipping, maturing, spawning and spent females by age (a) and total length (b) in *Melanogrammus aeglefinus* (NEA stock) during the sampling period. Letters a to c above the boxes indicate the compact letter display of significantly different groups in the two‐way ANOVA after a *post hoc* test for multiple comparisons of groups. Boxplots are in the style of Tukey (median = 50% quantile; upper and lower hinges = 75% and 25% quantile, respectively; whiskers ±1.5 × interquartile range). Closed circles, dots and transparent circles represent the mean, outliers and individual sample points, respectively

Within the material examined by histology, significant differences in age and TL existed among maturity categories (Figure 8), where maturing females were significantly older (two‐way ANOVA, *F*_4,287_ = 25.92, *P* < 0.001) and larger (*F*_4,293_ = 46.67, *P* < 0.001) (6.1 ± 2.2 years, 55.9 ± 9.5 cm) compared to immature (3.8 ± 0.8 years, 40.4 ± 7.1 cm) and skipping (4.9 ± 1.0 years, 48.9 ± 6.1 cm) females.

### 3.6 Proxies for energy storage

**FIGURE 9:**
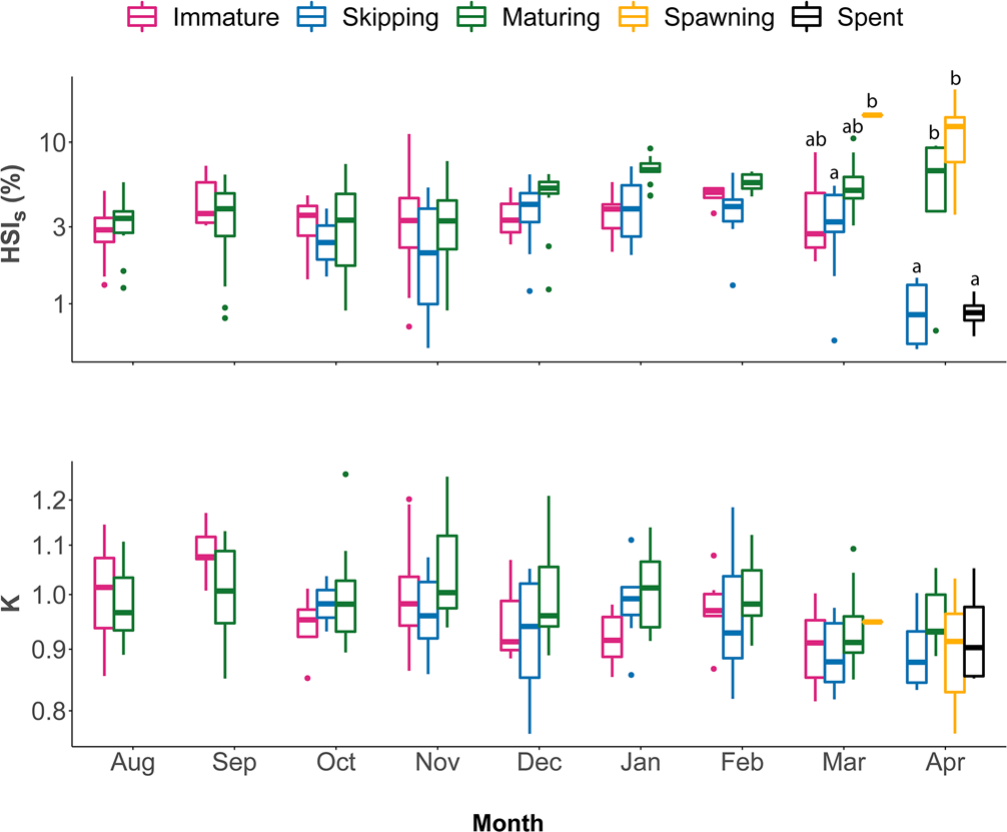
Energy proxies (a) somatic hepatosomatic index (HSI_S_) and (b) Fulton's condition factor (*K*) for all *Melanogrammus aeglefinus* (NEA stock) females histologically categorized as immature, skipping, maturing, spawning and spent over sampling months (August 2020 to April 2021). Letters a–c above the boxes indicate the compact letter display of significant different groups in the two‐way ANOVA after a *post hoc* test for multiple comparisons of groups. Boxplots are in the style of Tukey (median = 50% quantile; upper and lower hinges = 75% and 25% quantile, respectively; whiskers ±1.5 × interquartile range). Dots represent the outliers

Monthly changes in female energy storage, represented in the first instance by HSI_S_, appeared dependent on maturity category, where skipping females showed significantly lower values in March than spawning females (Figure 9a). In April, both skipping and spent females had a significantly lower HSI_S_ than spawning and maturing females (two‐way ANOVA, *F*_26,260_ = 8.13, *P* < 0.001; Figure 9a). For *K*, the interaction term *maturity category × month* was insignificant (*P* > 0.05), but the main effect, *i.e*., *maturity category + month*, was evidently in place (two‐way ANOVA, *F*_12,285_ = 5.17, *P <* 0.001; Figure 9b), where skipping females (November–April) showed a significantly lower *K* compared to maturing females (*P* = 0.004). Consequently, overall, HSI_S_ was a more sensitive parameter in these respects than *K*; *maturity category + month* explained 38.9% of the variability in HSI_S_ but only 14.4% in *K*.

## 4 DISCUSSION

The reproductive cycle of *M. aeglefinus* displayed several special features as a gadoid. Our histological analysis revealed an extended cortical alveoli phase, relative to the better‐studied *G. morhua*: in *M. aeglefinus* (NEA stock) CAO appeared as early as August (≈2 months earlier than cod; Kjesbu *et al*., 2011) and true vitellogenesis did not begin until December (≈2 months later than cod; Kjesbu *et al*., 2011). The currently estimated length of the spawning season of about 3 months agrees well with previous observations, but also that *M. aeglefinus* (NEA stock) is likely start releasing eggs in the order of 1 month later than *G. morhua* (NEA stock) (Bergstad *et al*., 1987). However, the extended CAO phase of *M. aeglefinus* is not unique in teleost. For example, this initial period of gonadotrophin‐dependent oocyte growth (Wallace & Selman, 1981) is known to last for at least 1 year in Antarctic toothfish (*Dissostichus mawsoni* Norman 1937) (Parker & Grimes, 2010) to several years in spotted wolffish (*Anarhichas minor* Olafsen 1772) (Gunnarsson *et al*., 2008). In Greenland halibut (*Reinhardtius hippoglossoides*; Walbaum, 1792) the CAO phase might persist throughout the whole reproductive cycle but under an exceptional reproductive style of two vitellogenic cohorts ultimately splitting apart supporting successive annual spawning (Kennedy *et al*., 2011). However, these outlined patterns of an extended CAO phase are not necessarily controlled by low biochemical activity in cold waters as *G. morhua* (NEA stock) females proceed directly from late PVO to CAO (Kjesbu *et al*., 2011, 2010), provided not being skippers. The reason why *M. aeglefinus* would progress into the CAO phase earlier than *G. morhua*, despite entering vitellogenesis later and subsequently spawning later in the spring, appears evasive. Still, the first appearance of EVO in the present study (October 27) resembles that of a recent study of the North Sea saithe (*Pollachius virens*; L. 1758) (Skjæraasen *et al*., 2017), showing initiation of vitellogenesis in late October–early November, nearly a month later than in cod. Skjæraasen *et al*. (2017) elaborated on whether the laboratory settings associated with their study could have shifted the timing of vitellogenesis, but along with the results from the present study, this discrepancy could indicate potential real species differences in the onset of vitellogenesis between gadoids.

In the months close to the start of the spawning season, the considerable variability in oocyte development across *M. aeglefinus* (NEA stock) individuals was evident in that some females, at a given day, showed up to 300 μm larger LC sizes compared to others. The reason for the observed and extrapolated variability in spawning time was partly related to size‐specific effects (Tobin *et al*., 2010; Wright & Trippel, 2009), with the present simulation suggesting the start of spawning at TL = 70 cm about 3 weeks earlier than at TL = 40 cm. Such a size effect on LC could potentially be related to the fact that immature females developing for their first spawning season (recruit spawners) may start the formation of CAO later in the autumn compared to already sexually mature females (repeat spawners), as observed in both *M. aeglefinus* in the North Sea (Tobin *et al*., 2010) and *G. morhua* (Kjesbu *et al*., 2011). Size‐specific differences in LC are generally found in *G. morhua* (Kjesbu, 1994; Kjesbu *et al*., 2010; Skjæraasen *et al*., 2010) but have not previously been documented for *M. aeglefinus* (NEA stock) (Skjæraasen *et al*., 2013), and may thus be a contributing factor to the long CAO phase observed in the present study.

In this tracking study we were for the first time able to demonstrate that the ultimate cause behind reproductive interruption of *M. aeglefinus* is massive atresia of CAOs. Such extensive reabsorption has earlier been reported both in field‐caught and experimentally monitored *G. morhua* (Rideout, 2000; Skjæraasen *et al*., 2009). The first evidence of this fine‐tuned mechanism was presently observed as early as October–November. Hence, examinations of samples originating from later in the reproductive cycle should in principle be unable to cover the full picture. Skjæraasen *et al*. (2015) reported resting, *i.e*., skipping females that do not advance beyond PVO phases, as being the dominant mode (95.6% up to 98.8% in 2009–2012) during the months from February to March, with the remaining trivial proportions of skipping females classified as either reabsorbing‐CAO or ‐VO. Consequently, the resting‐PVO mode should be expected to specifically refer to the end (closure) of the reabsorbing‐CAO mode, *i.e*., following completed CAO atresia, indicated presently by the gradual accumulation of resting skippers and the associated declining proportion of reabsorbing skippers. This way of thinking markedly differs from the conventional one, where resting skippers are said to not advance beyond PVO phases (see Introduction). We therefore claim that an individual *M. aeglefinus* becoming a skipper enters the CAO phase, as default, but becomes physiologically arrested at the following energetically demanding vitellogenin sequestration. Both steps are part of the gonadotrophin‐dependent oocyte growth, indicating that the animal is sexually mature (Lubzens *et al*., 2017; Wallace & Selman, 1981).

The above findings at the oocytic level suggest that insufficient energy reserves might trigger skipping of the reproductive cycle in *M. aeglefinus* (NEA stock). Compared to *G. morhua* (NEA stock), *M. aeglefinus* (NEA stock) not only show approximately 40% higher fecundity, selecting TL = 60 cm as the overlapping reference point (Kjesbu *et al*., 1998; Skjæraasen *et al*., 2013), but also typically prey on Echinodermata (Jiang & Jørgensen, 1996; Tam *et al*., 2016) instead on highly energy‐rich fishes such as capelin (*Mallotus villosus*; Müller 1776) (Holt *et al*., 2019). Thus, one should expect a relatively higher energetic strain. However, the results from the present study did not find any clear evidence that HSI_S_ or *K* was significantly lower at the time of reproductive commitment in skipping females (late October–early November), although a nonsignificant trend was noticed. Nonetheless, skipping females generally exhibited lower values of HSI_S_ and *K* compared to maturing females. High variability in *K* and particularly HSI_S_ was common, indicating that other factors than maturity status or temporal variability may have affected these parameters. Alternatively, reproductive decisions were potentially being made much earlier, following a critical post‐spawning feeding period (Burton, 1994; Lambert & Dutil, 1997; Rideout *et al*., 2005). Skipping females in the present study were on average ~5 years old and 49 cm long, indicating that a considerable proportion of females that had completed their first spawning season as 4‐year‐olds (Olsen *et al*., 2010) would skip their next spawning opportunity. A possible explanation for this observation could be that recruit spawners may have difficulties in acquiring enough energy following their first spawning season to repeat the development process for next year's spawning opportunity. The interruption of maturity prior to the onset of vitellogenesis and the start of the lengthy spawning migration seem to be highly beneficial to conserve and divert energy into other physiological processes. Demographic effects of skipped spawning have also been demonstrated previously in *M. aeglefinus* (NEA stock) (Skjæraasen *et al*., 2015) and other gadoids (Macchi *et al*., 2016; Skjæraasen *et al*., 2012), suggesting links to both energy constraints and age diversity. No skipping females were detected among females above 8 years and 64 cm, indicating that older, larger females generally spawn annually, investing energy into reproduction rather than body growth.

Although it is beyond the scope of the present individual study to scale up to population dynamics and the associated overall levels of skipping across year (Skjæraasen *et al*., 2020), one might wonder how *M. aeglefinus* (NEA stock) will do in the future under ongoing climate change. A recent climate impact study suggests that this stock will develop positively under the intermediate RCP 4.5 climate scenario until 2050, in stark contrast to *M. aeglefinus* (North Sea), due to increased gross primary and secondary production and less sea ice abundance in the Barents Sea (Kjesbu *et al*., 2022). Increasing bottom temperature is largely counteracted by behavioural overcompensation, *i.e*., moving into ambient colder waters even though the environmental temperature as such increases (Landa *et al*., 2014), a phenomenon seen in many species able to move polewards or into a colder ocean current branch (Kjesbu *et al*., 2022).

To conclude, the present study has highlighted important new processes related to the reproductive cycle of *M. aeglefinus* (NEA stock). These results have implications for future reproductive studies of *M. aeglefinus* by in‐depth characterization of continuous oocyte growth of maturing individuals and terminated oocyte development of skippers. It would be beneficial if *M. aeglefinus* (NEA stock) ovary samples in future studies were collected earlier in the reproductive cycle (*e.g*., immediately after the spawning season) to assess the nutritional status of females at a time when the physiological decision to spawn might be predetermined.

## AUTHOR CONTRIBUTIONS

This contribution is a revision of an earlier Master of Science thesis in Fisheries Biology and Management, University of Bergen, Norway, defended by F.T in June 2021. E.J., O.S.K. and F.T. conceived the study. O.S.K designed the laboratory analysis. F.T. and M.A. processed samples in the laboratory. F.T., T.C.S.S. and K.C. analysed the data. F.T., O.S.K and E.J. wrote the manuscript. A.F provided supervision. E.J. and O.S.K acquired funding. All authors commented on manuscript drafts and approved the final version of the manuscript.
